# Insights into the aroma volatiles and the changes of expression of ester biosynthesis candidate genes during postharvest storage of European pear

**DOI:** 10.3389/fpls.2024.1498658

**Published:** 2024-11-29

**Authors:** Xinxin Zhu, Xiaofei Xu, Fudong Jiang, Qingyu Li, Aidi Zhang, Jianzhao Li, Hongxia Zhang

**Affiliations:** ^1^ The Engineering Research Institute of Agriculture and Forestry, Ludong University, Yantai, China; ^2^ Yantai Academy of Agricultural Sciences, Yantai, China; ^3^ School of Food Engineering, Ludong University, Yantai, China; ^4^ Shandong Institute of Sericulture, Shandong Academy of Agricultural Sciences, Yantai, China; ^5^ Zhaoyuan Shenghui Agricultural Technology Development Co., Ltd, North of Beiyuanzhuang village, Fushan County, Zhaoyuan, Shandong, China

**Keywords:** pear, volatile compounds, postharvest, aroma, gene

## Abstract

During the storage period after harvest, the presence of volatile esters is essential for European pear aroma. Nevertheless, the specific molecular process underlying the production of volatile esters in European pear remains elusive. In this research, head space solid phase microextraction and gas chromatography-mass spectrometry were employed to examine the volatile compounds of two varieties of European pear. The results revealed the identification of a collective of 149 volatile compounds, which were categorized into 8 groups: esters (37), alcohols (25), alkanes (24), aldehydes (22), terpenes (15), acids (8), ketones (6) and other categories (12). Notably, there were 79 volatile compounds that coexisted in both varieties, which esters are the primary group of volatile compounds found in both varieties. Through transcriptome analysis, we identified 12 candidate genes associated with ester biosynthesis and established their correlation with firmness, ethylene production, and predominant volatile esters. The results from gene expression analysis revealed significant up-regulation of *PcFAD2* and *PcLIP2* in both varieties and *PcFAD6* exhibits low expression levels. The results indicate that the involvement of these three genes in the synthesis of esters in European pear may have a significant level of importance. This study enhances our understanding of the mechanisms involved in the formation of European pear flavor.

## Introduction

The European pear is one of the two types of cultivated pear in the world, which is as famous as the Oriental pear. It was first introduced by Yantai in China and has now become one of Yantai’s characteristic fruits. European pear is a typical fruit variety characterized by a respiratory jump. When first harvested, the fruit is firm, and the flesh exhibits a light and aromatic quality. With the extension of room temperature storage time, the fruit gradually ripens, resulting in delicate and juicy flesh accompanied by a strong aroma that appeals to consumers (Silva et al., 2014; Wang et al., 2024). Yantai predominantly cultivates the European pear varieties known as ‘Doctor Jules Guyot’ and ‘Red Clapp Favorite’. They are early maturing varieties, ripening around August. The aroma of ‘Doctor Jules Guyot’ pear becomes rich after ripening, while ‘Red Clapp Favorite’ pear has a poor aroma and even carries an alcoholic taste. In the past few years, individuals’ living standards and consumption levels have witnessed a remarkable enhancement, leading to an increasing desire among consumers for European pears of superior quality. The increasing body of research indicates a positive association between fruit aroma and consumer acceptance (Moya-León et al., 2006; Delgado et al., 2013; Fan et al., 2021; Ferrão et al., 2022). Fruit aroma is the combination of the interaction of volatile and non-volatile substances in fruit that work together (Jia et al., 2023). The unique fragrance of every fruit is determined by its distinctive combination of volatile substances (Yao et al., 2023). Although consumers first come into contact with the peel when purchasing fruit, the main edible part of European pears is the flesh, and its internal quality affects consumer experience (Xin et al., 2018; Liu et al., 2020). This is the important factor that attracts consumers to purchase more frequently. Moreover, scientific research has demonstrated that the pigmentation of fruit can significantly influence the production of volatile compounds within them, consequently impacting their aroma (Xin et al., 2018; Rodríguez-Lorenzo et al., 2023).

The volatile compounds found in fruit have been extensively studied, included apple (Ulrich and Dunemann, 2012), peach (Wang et al., 2009), orange (Aubert and Pitrat, 2006) and mango (Lalel et al., 2003). The analysis of pears has identified over 300 distinct volatile compounds to date (Rapparini et al., 2008). These compounds include esters, aldehydes, alcohols, and more. Among them, volatile esters are essential in determining the fragrance of matured European pear fruit (Qin et al., 2012). Variations in the volatile compounds, both in terms of their types and quantities, contribute to the distinct aromas observed among different European pear cultivars (Farré-Armengol et al., 2017). The European pear varieties ‘La France’, ‘Alexandrine Douillard’ and ‘Beurre Bosc’ are known for their high ester content, resulting in a robust ester scent. In contrast, the ‘Bartlett’ variety exhibits a lower ester concentration but possesses a distinct flavor (Chen et al., 2018).

The fatty acid serves as a crucial precursor for the synthesis of fruit aromatic compounds (Jiang et al., 2022). It undergoes catalytic conversion by fatty acid desaturase (FAD) and lipase (LIP) to yield fatty acids with unsaturation, including linolenic acid and linoleic acid (Liu et al., 2018; Luo et al., 2021a; Cao et al., 2023). The volatile esters in aroma components primarily originate from the metabolic breakdown of fatty acids through oxidation (Qin et al., 2014), encompassing both the lipoxygenase (LOX) pathway and β-oxidation (Liu et al., 2023). In the β-oxidation pathway, esters are formed from saturated fatty acids through a series of sequential acetyl-coa eliminations, resulting in the formation of various acyl coa molecules. In the LOX pathway, lipoxygenase facilitates the enzymatic conversion of unsaturated fatty acids into hydroperoxides (Feussner and Wasternack, 2002). Subsequently, hydroperoxide lyase (HPL) converts these hydroperoxides into aldehydes (Mita et al., 2005), which are then Converted into their respective alcohols through the action of alcohol dehydrogenase (ADH). Finally, alcohol acyl transferase (AAT) further reduces the alcohols to produce various ester aroma substances (Beekwilder et al., 2004; Li et al., 2014; Chen et al., 2016; Wei et al., 2016; Luo et al., 2021a).

Recent research findings have indicated that the involvement of LOX pathway enzyme synthesis genes is pivotal in the development of fruit aroma. For instance, the expression of *PpFAD2-1* exhibited an upward trend as peach fruit matured, and its overexpression significantly enhanced the accumulation of aromatic compounds in peach fruit after being subjected to cold storage (Peng et al., 2023). Similarly, the overexpression of *SlFAD3/7* promotes volatile accumulation in tomato fruit and contributes to fruit aroma development (Domínguez et al., 2010). *MdAAT1* and *MdAAT2* have demonstrated their involvement in ester synthesis in apples (Li et al., 2023). Furthermore, ethylene and ABA treatment can upregulate *MiADH1* in mangoes, highlighting its significance in fruit aroma volatiles (Singh et al., 2018). However, during the postharvest storage of European pear, the composition and synthesis pathway of volatile esters become intricate and the encoding genes responsible for key enzyme activities remain largely unknown (Xie et al., 2013). Therefore, further investigation into gene expression during postharvest storage is necessary for a comprehensive understanding of European pear aroma formation.

Based on the above background, ‘Doctor Jules Guyot’ and ‘Red Clapp Favorite’, two exceptional cultivars of European pear, were selected as the subjects of investigation. Initially, headspace solid phase microextraction (SPME) and gas chromatography-mass spectrometry (GC−MS) were employed to quantitatively analyze the aroma compounds in the flesh of pear fruit and compare disparities in composition and content of volatile substances among different varieties. Subsequently, transcriptome data were utilized for further investigation to identify candidate genes responsible for regulating ester biosynthesis from a pool of numerous genes. The levels of expression for these potential genes in comparison to each other during postharvest storage were determined to ascertain whether they are regulated during fruit preservation and their impact on aroma formation. The objective of this study is to accumulate data for future comprehensive research on the fragrance characteristics of European pear.

## 2 Materials and methods

### 2.1 Plant material

Pear fruit samples for this study were collected on July 24, 2020 at Yantai Academy of Agricultural Sciences in Shandong Province, China (latitude: 37.4893; longitude: 121.2790) through manual collection. The collected varieties included ‘Doctor Jules Guyot’ and ‘Red Clapp Favorite’, which are European pear varieties. At least 30 fruit trees were randomly selected for each variety. The minimum requirement for each fruit tree is to have a random selection of at least 20 fruit and fruit were gathered on the identical day and quickly transported to the laboratory, where they underwent rigorous screening. The screened fruit were stored in a temperature-controlled chamber at 25°C and 80% humidity. Subsequently, nine fruit were randomly selected every five days for sampling in order to determine relevant physiological indexes. The measurement method of firmness and ethylene were determined according to the method proposed by Dai et al. (2023). Due to the significant difference in color of the peel between two European pear varieties, this study only selected the milky white flesh of these two varieties. The flesh was chopped into sealed bags and numbered before being stored at -80°C for subsequent analysis purposes. Each experimental group was comprised of three biological replicates, with each replicate containing three fruit (Dai et al., 2023).

### 2.2 Volatile analysis by GC-MS

European pear volatiles were conducted in accordance with Zhang et al. (2020). European pear flesh is transformed into a powdered form by employing the technique of liquid nitrogen freezing. A 2.0 g portion was precisely measured and introduced into a glass bottle with a capacity of 15 mL, accompanied by the addition of 3 mL methanol and 10 µL solution containing 2-octanol at a concentration of 0.8 mg/mL. Among them, an internal standard of 2-octanol was employed. Each sampling point has three replicates. After vortex mixing, the mixture was equilibrated at 45°C for 30 min, and the volatile compounds were collected using solid phase microextraction (SPME) fiber, which was 50/30 µm DVB/CAR/PDMS (USA). The Shimadzu GC-2030 GC column chromatograph, in conjunction with the Shimadzu GCMS-QP2020 NX mass spectrometer, was utilized for the identification of volatile compounds. The column chromatograph employed a SH-Stabilwax column (60 m length, 0.25 mm inner diameter, and 0.25 µm film thickness). The temperature protocol commenced with an initial temperature of 40°C, gradually escalating to 100°C at a pace of 3°C per minute. Subsequently, it further ascended until reaching a final temperature of 230°C, progressing at a rate of 5°C per minute. The carrier gas used was helium with a flow rate set at 1.0 mL min^-1^. The source temperature of the mass spectrum was set to 230°C, the electron energy of the column out let was 70 eV, and the transfer interface temperature was 250°C. Qualitative scanning was performed in the range of 35-350 m/z. Initial identification of volatile compounds was achieved by comparing their electron ionization mass spectra with those from the NIST-2017 mass spectra library.

### 2.3 RNA extraction, cDNA synthesis and RNA seq

The total RNA was extracted from European pear fruit samples using an RNA extraction kit (Tiangen, Beijing, China) and operated according to the specifications. The concentration and purity of the resulting RNA samples were assessed using an NP80Touch ultramicrospectrophotometer (Implen, Germany). Subsequently, cDNA synthesis was performed utilizing the HiScript III-RT SuperMix for qPCR reverse transcription kit (Vazyme, Nanjing, China). The construction of the RNA library was based on previous sequencing results conducted by our research group (Dai et al., 2023). Illumina HiSeq X platform was employed for sequencing. Each time point in the RNA-seq analysis consisted of three biological replicates.

### 2.4 Real-time PCR analysis

Quantitative real-time PCR analysis (qRT-PCR) and the 2^−ΔΔCt^ method were used to determine the transcript abundance of 12 ester biosynthesis genes in ‘Doctor Jules Guyot’ during storage. qRT-PCR was performed using the Vazyme kit ChamQ Universal SYBR qPCR Master Mix kit (Nanjing, China) and a Bio-Rad CFX Connect system. All reactions were carried out in duplicate in 96-well plates. The qRT-PCR procedure followed the following steps: 30 s at 95°C, followed by 40 cycles of 5 s at 95°C and 30 s at 60°C. Subsequently, additional cycles were performed to construct the dissolution curves. The cDNA was diluted to a Ct value of around 20 prior to qRT-PCR. For real-time fluorescent quantitative PCR analysis of gene primers designed using primer3 (http://frodo.wi.mit.edu/primer3)(**Supplementary Table 1**).

### 2.5 Statistical analysis

The heat map was generated using TBtools software. Correlation analysis was plotted using Origin 2022 (USA). The other images were drawn by GraphPad Prism 9 software. Statistical significance was assessed using a t-test (*P < 0.05; **P < 0.01; *** P < 0.001). The experimental data were generated from three repeated experiments.

## 3 Results

### 3.1 Volatile compounds during postharvest storage of two varieties

**Figure 1:**
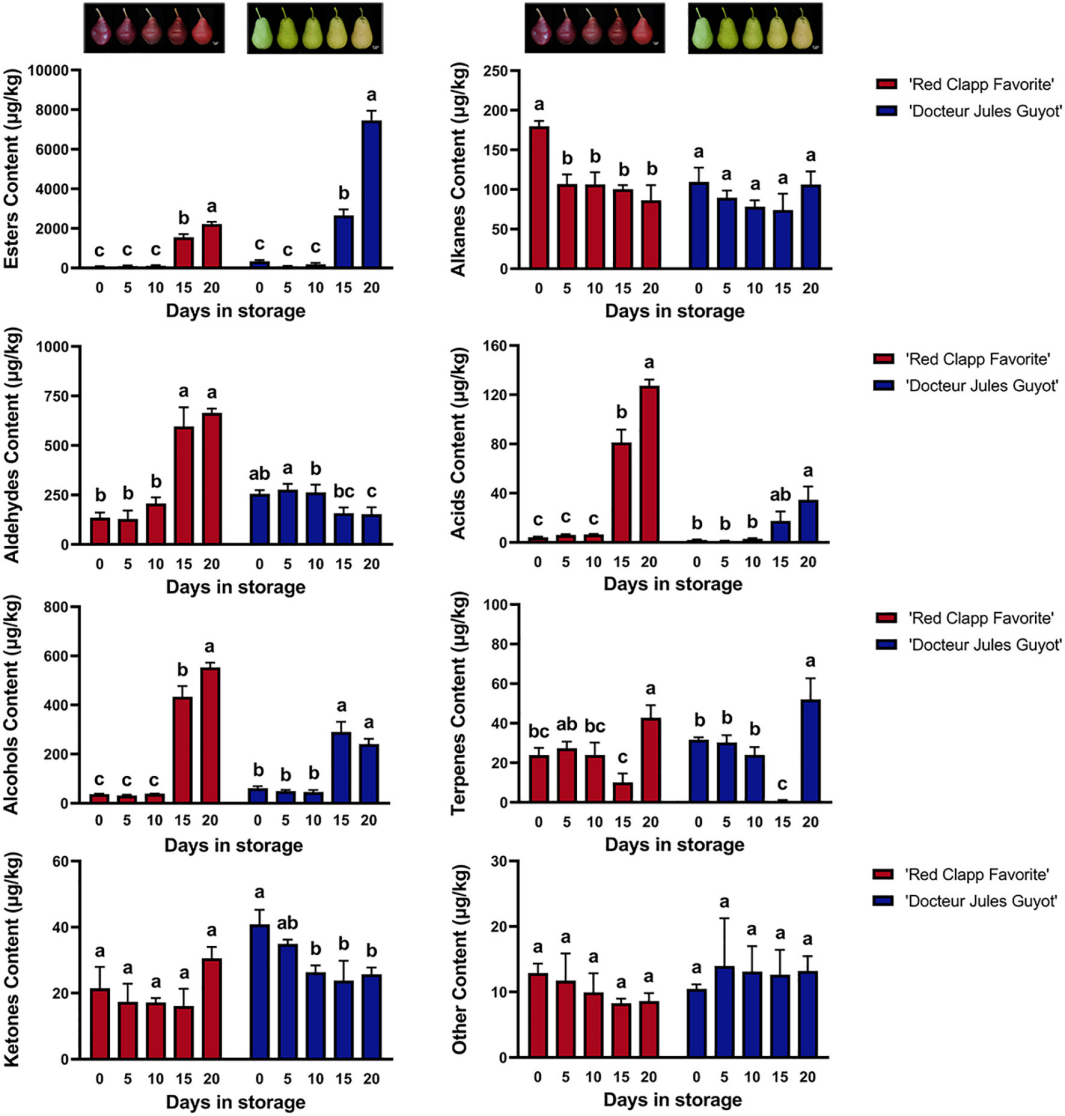
The fruit images and the total volatile compound content of ‘Red Clapp Favorite’ and ‘Doctor Jules Guyot’ at different postharvest storage stages. The concentration is represented as 2-octanol equivalent concentration. Error bars represent ± SE (n=3). Analysis of variance (ANOVA) conducted using SPSS indicated that the observed differences between letters **(A-C)** at P < 0.05 were statistically significant.

The volatile compounds of European pear ripe fruit were subjected to analysis by using GC-MS, and 149 different types of volatile compounds were detected (**Supplementary Table 2**). These compounds can be categorized into eight chemical classes: esters, alkanes, aldehydes, acids, alcohols, terpenes, ketones, and other unclassified compounds (**Figure 1**). Among these groups, esters are the most abundant with a total of 37 different types present in European pears. This is followed by alcohols and alkanes which consist of 25 and 24 different types, respectively. A comprehensive analysis of ‘Doctor Jules Guyot’ revealed the presence of 116 volatile compounds, comprising 31 esters and 20 alcohols. In ‘Red Clapp Favorite’, a total of 109 volatiles were detected, which was slightly lower than that in ‘Doctor Jules Guyot’, and the number of alkanes and esters was higher with 20 and 19, respectively. Notably, significant variations in both type and quantity of volatile compounds were found in the fruit flesh of European pears during postharvest storage time as well as between different varieties. With the increase of storage time, some specific types of volatile compounds may show a significant increase or decrease trend when comparing different varieties.

Although in both varieties, the accumulation of alcohols and acids consistently increases over time during storage. However, when the storage time reached 20 days, the total alcohol content of ‘Red Clapp Favorite’ was 2.29 times higher than that of ‘Doctor Jules Guyot’, and the total acid content was 3.66 times higher than that of ‘Doctor Jules Guyot’. The concentration of aldehydes exhibited a decreasing trend over time in the cultivar ‘Doctor Jules Guyot’, whereas it showed an increasing pattern in the variety ‘Red Clapp Favorite’. Additionally, there was no notable variation in alkane levels in ‘Doctor Jules Guyot’ with increasing storage time, whereas the amount of alkane significantly decreased in ‘Red Clapp Favorite’. It can be observed that the substantial increase in alcohols, aldehydes, and acids in the ‘Red Clapp Favorite’ variety, along with a decrease in alkanes, may indicate its production of unique volatile compounds during storage which contribute to its distinct aroma.

Interestingly, the ester content in both varieties exhibited a rapid increase during the later stage of storage, surpassing that of other compounds. Ester compounds are widely recognized as crucial volatile components in European pear. Specifically, after 20 days of storage, the ester content of the ‘Doctor Jules Guyot’ variety reached 22390 μg/kg, which was significantly higher compared to that of the ‘Red Clapp Favorite’ variety, exhibiting a content 3.35 times greater. Surprisingly, only ester compounds in ‘Doctor Jules Guyot’ were found to be much higher in late storage than in ‘Red Clapp Favorite’ varieties. In addition, the levels of ketones, terpenes, and other volatile compounds remained relatively stable during postharvest storage.

### 3.2 Comparison of main volatile compounds between two varieties

**Figure 2:**
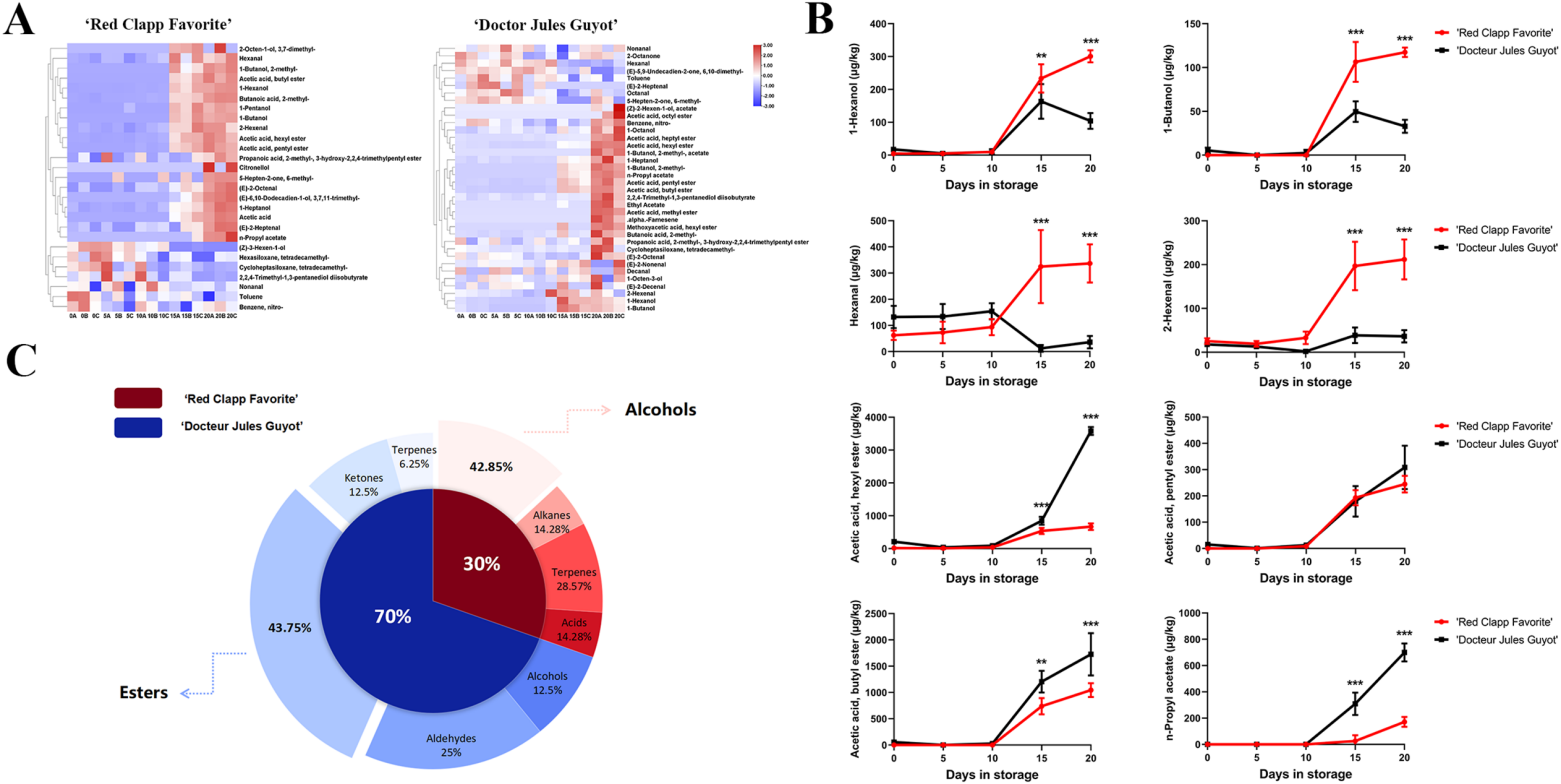
Comparison of the main volatile compounds between ‘Red Clapp Favorite’ and ‘Doctor Jules Guyot’. **(A)** Heat map demonstration showcasing the primary volatile compounds found in each species. **(B)** Line diagrams depicting the primary volatile compounds that are common to both varieties. **(C)** The number of volatile compounds unique to each species. Error bars represent ± SE (n=3). (**P < 0.01, and ***P < 0.001).

In a comprehensive analysis of volatile compound data for ‘Doctor Jules Guyot’ and ‘Red Clapp Favorite’, we initially excluded data with poor repeatability and compound content below 15 μg/kg throughout the postharvest storage period. TBtools software was employed to analyze the cluster heat map of the processed data, revealing the temporal variation trend of volatile compounds in European pear during postharvest storage. On the whole, the volatile compounds of the two varieties are classified into two distinct categories. Compounds exhibiting a positive correlation between their content and storage time increase are grouped together, while those with a negative correlation are placed in another category (**Figure 2A**). Additionally, after data screening, it was observed that ‘Doctor Jules Guyot’ contains more volatile compounds compared to ‘Red Clapp Favorite’. From a classification perspective, the majority of esters, aldehydes, and alcohols in both varieties showed an increasing trend with storage time, including hexyl alcohol, butanol, heptanol, 2-hexenal, butyl acetate, hexyl acetate and others. Conversely, the content of alkanes and ketones decreased as storage time increased. However, it is worth noting that in both varieties there were significantly more positively correlated volatile compounds than negatively correlated ones.

Further analysis of the aforementioned hypothesis led to the identification of common volatile compounds in both varieties. Compounds with a content below 100 μg/kg throughout the entire postharvest storage process were excluded, resulting in the identification of eight volatile compounds: hexyl alcohol, butanol, hexaldehyde, 2-hexenoyl aldehyde, hexyl acetate, amyl acetate, butyl acetate and n-propyl acetate (**Figure 2B**). It is evident that the levels of these eight volatile compounds gradually increase as postharvest storage time progresses. Notably, when reaching a storage time of 10 days, ‘Doctor Jules Guyot’ exhibited significantly lower contents of hexyl alcohol, butanol, hexaldehyde and 2-hexenal compared to ‘Red Clapp Favorite’. Conversely, during later stages of storage, ‘Doctor Jules Guyot’ displayed significantly higher contents of hexyl acetate, amyl acetate, butyl acetate and n-propyl acetate than ‘Red Clapp Favorite’. It is important to mention is that these four compounds which are significantly higher in ‘Doctor Jules Guyot’ are ester compounds and their growth rate is extremely fast.

Additionally, we identified volatile compounds specific to each variety and excluded compounds with a content below 15 μg/kg throughout the postharvest storage process. The results were presented in pie charts indicating the number of species (**Figure 2C**). The figure illustrates that ‘Doctor Jules Guyot’ contains a higher concentration of unique substances compared to ‘Red Clapp Favorite’, accounting for 70% of the total unique substances, with esters being the main component, accounting for 43.75% of the total unique substances in ‘Doctor Jules Guyot’.

### 3.3 Expression patterns of ester biosynthesis related genes during postharvest storage

**Figure 3:**
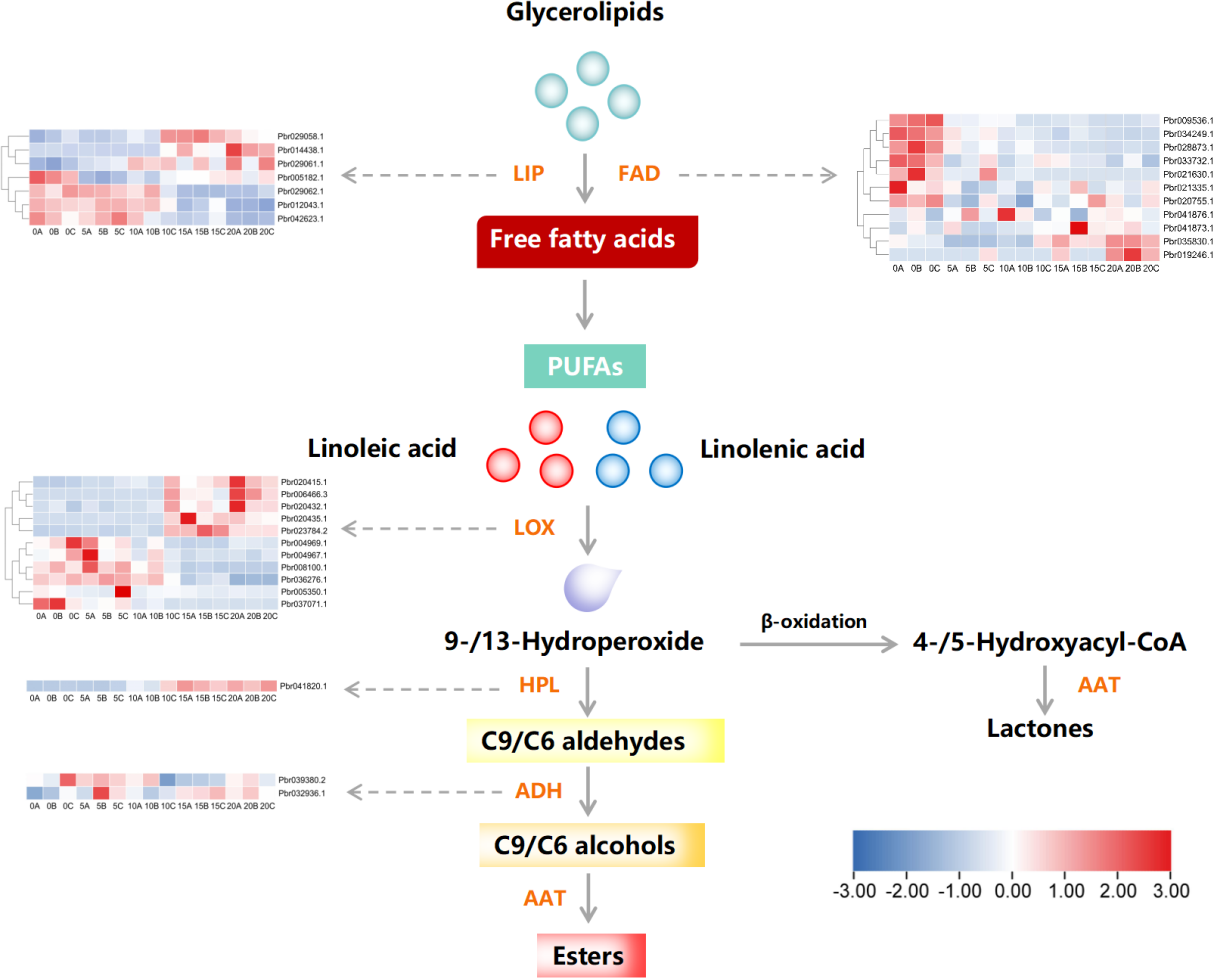
Pathway examination of genes associated with the production of esters in European pear during storage after harvest. The heat map illustrates the expression pattern of individual genes. FAD, fatty acid desaturase; LIP, Lipase; LOX, lipoxygenase; HPL, hydrogen peroxide lyase; ADH, alcohol dehydrogenase; AAT, alcohol acyltransferase. The horizontal axis of the heat map represents the storage time after harvesting, with numbers indicating the number of days and letters A, B, and C representing three repetitions.

The obtained results so far emphasize the potential role of esters in aroma formation of European pear. To further investigate the impact of esters on aroma formation at the transcriptional level, we conducted heat map analysis based on transcriptome analysis results to study gene expression patterns involved in ester biosynthesis (**Figure 3**). We have excluded genes with FPKM values of 0 at various time points during post-harvest storage. As depicted in the figure, a total of 32 genes related to ester biosynthesis were identified, including 11 LOXs, 1 HPL, 2 ADHs, 11 FADs, and 7 LIPs. The genes were predominantly grouped into two major clusters, with cluster 1 exhibiting a significant temporal increase during postharvest storage, whereas cluster 2 demonstrated a notable decline.

### 3.4 Correlation of ester biosynthesis candidate genes, ethylene, firmness, and major ester components

**Figure 4:**
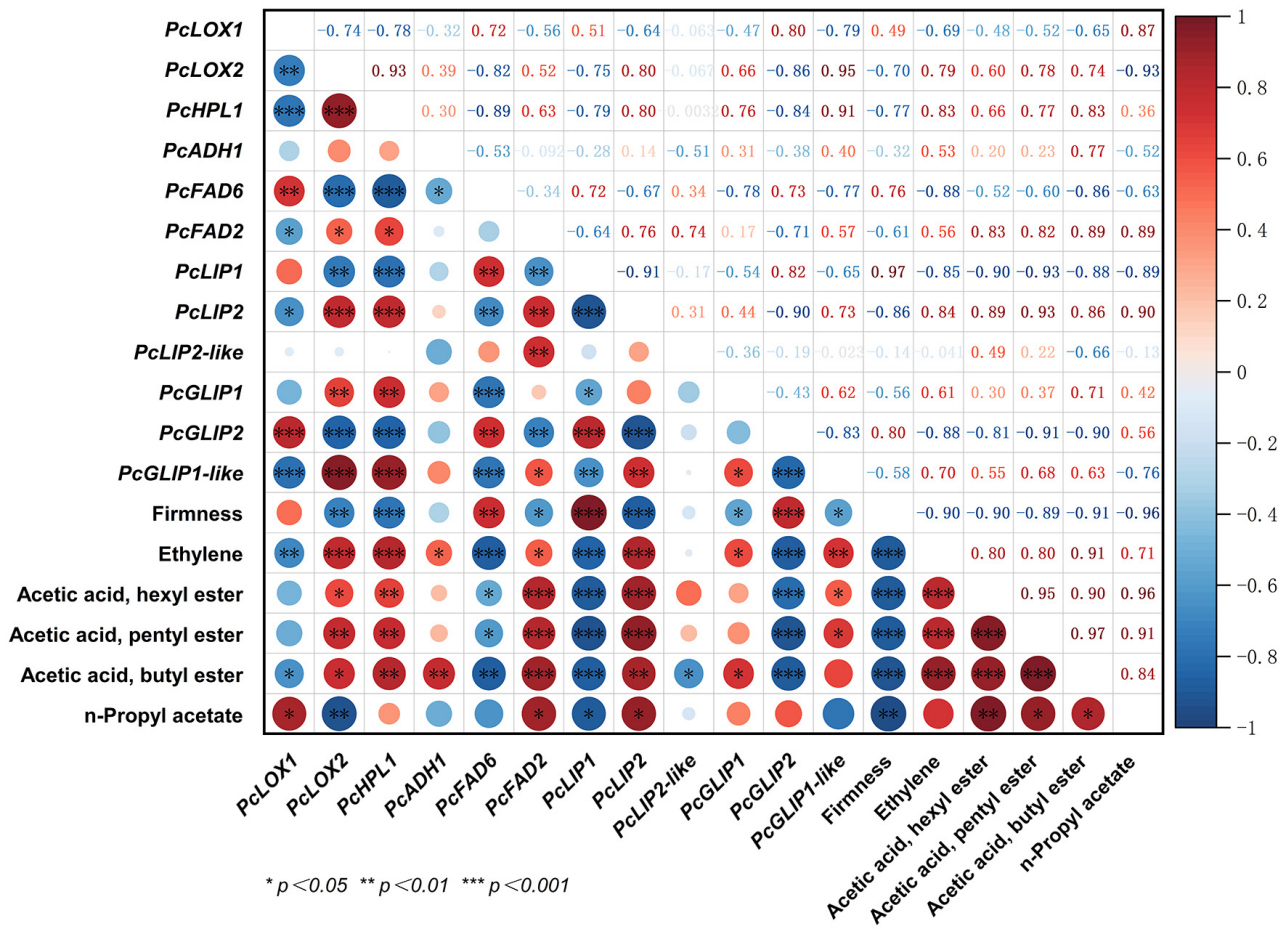
Correlation analysis of 12 ester biosynthesis candidate genes with firmness, ethylene and major volatile esters of European pear. The color scheme employed in this study utilizes a dark red hue to signify a positive correlation, while employing a deep blue shade to indicate a negative correlation. The magnitude of the circle is directly proportional to the strength of the observed correlation. (*P<0.05, **P < 0.01 and ***P < 0.001).

The European pear exhibits typical climacteric characteristics, including a decline in fruit firmness and an elevation in ethylene production throughout the postharvest storage period. The above 32 genes involved in ester biosynthesis undergo further screening to eliminate genes with low reproducibility and FPKM values below 20, and the remaining genes were correlated with firmness, ethylene production, and the four esters with the highest abundance in European pears. The results were visualized using Origin 2022 software (**Figure 4**). As depicted in the figure, we have identified a total of twelve genes, among which seven genes showed significant positive correlation with ethylene production, while four genes exhibited significant negative correlation. Notably, *PcFAD2* and *PcLIP2* displayed significant positive correlation with both four esters and ethylene production, whereas *PcFAD6* and *PcLIP1* demonstrated significant negative correlation.

### 3.5 Identification of candidate genes involved in ester biosynthesis in ‘Doctor Jules Guyot’

**Figure 5:**
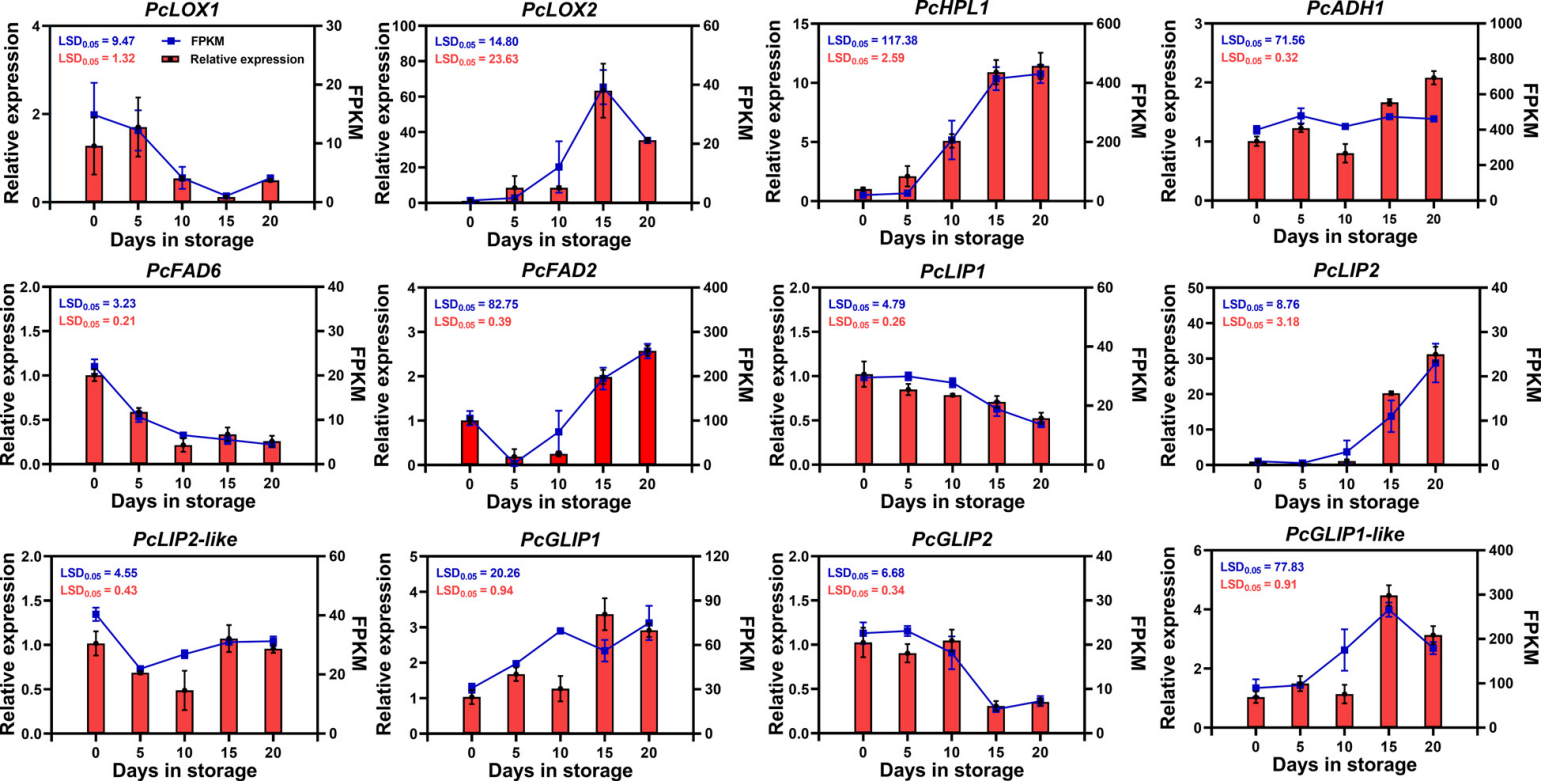
qRT-PCR analysis of the expression patterns of 12 candidate genes involved in ester biosynthesis in ‘Doctor Jules Guyot’ during postharvest storage. The relative expression levels determined by qRT-PCR are represented on the columns (left Y-axis), while RNA-seq expression levels in FPKM values are shown on the lines (right Y-axis). Error bars indicate ± SE (n = 3). The LSD value represents a significance level of p=0.05.

We validated the expression of these 12 candidate genes in ‘Doctor Jules Guyot’ fruit at the postharvest storage stage by using qRT-PCR. The results exhibited a high degree of concordance with the transcriptome data. The correlation coefficient between RNA-seq and RT-qPCR results exhibited a remarkably high level of significance, measuring 0.9759, thereby further substantiating the reliability of the transcriptome data. (**Figure 5**; **Supplementary Figure S1**). qRT-PCR analysis revealed that the 12 candidate genes could be categorized into four distinct expression patterns. Among them, *PcHPL1*, *PcADH1*, *PcFAD2*, *PcLIP2* and *PcGLIP1* displayed an upward trend in expression levels as postharvest storage time increased. Notably, both *PcHPL1* and *PcLIP2* showed a rapid increase in expression by 2.1-fold and 17.5-fold respectively when stored for 10-15 days. In the storage period of fifteen days, the expression levels of *PcLOX2* and *PcGLIP1-like* genes reached their peak, increasing by 57.5-fold and 4.3-fold compared to day 0, respectively. However, between day 15 and day 20, the expression levels of these two genes showed a decline. Additionally, *PcFAD6*, *PcLIP1* and *PcGLIP2* demonstrated decreased expression over time. Finally, the expressions of both *PcLOX1* and *PcLIP2-like* remained relatively stable.

### 3.6 Validation of candidate genes involved in ester biosynthesis in ‘Red Clapp Favorite’

**Figure 6:**
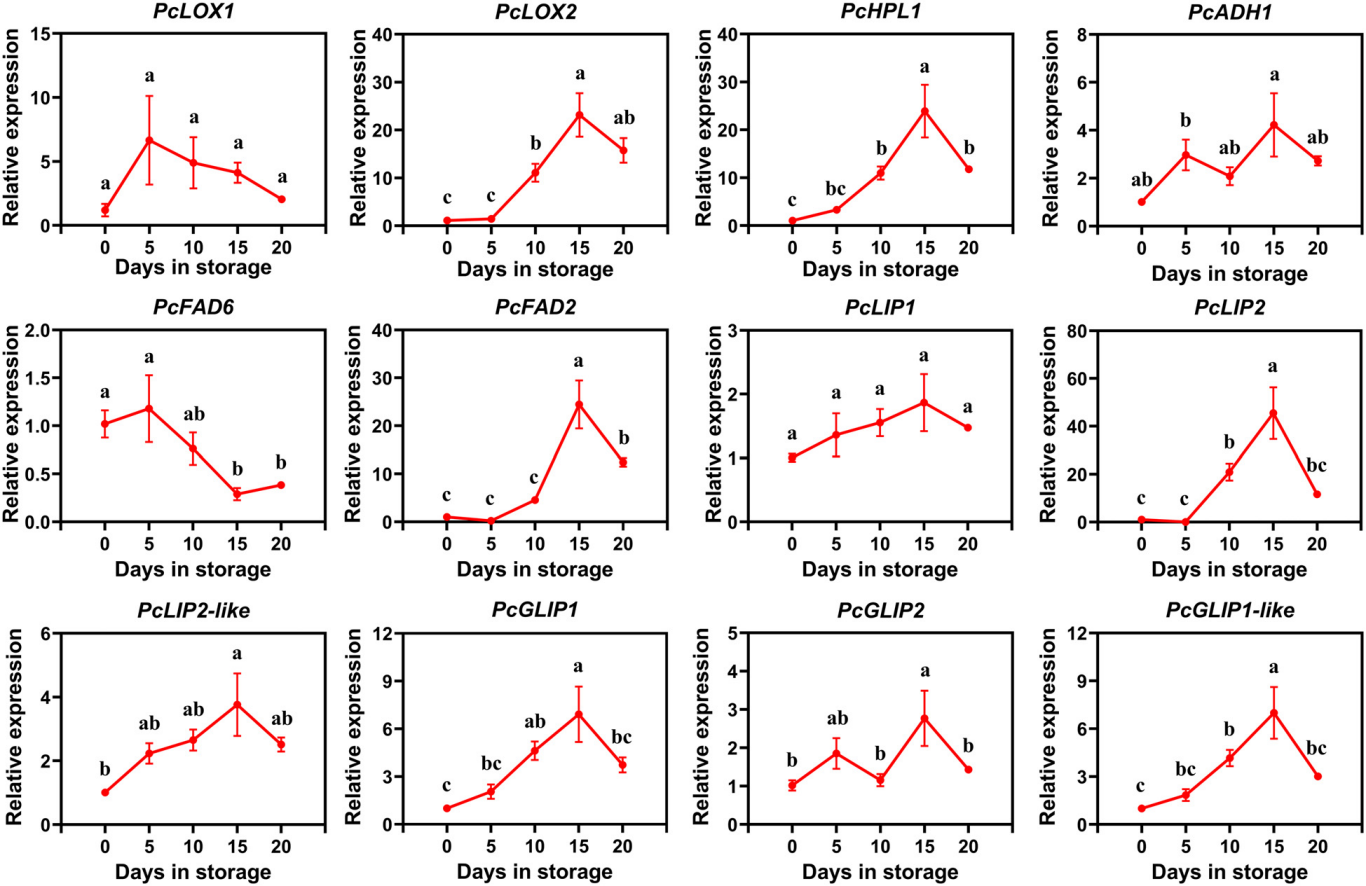
qRT-PCR analysis of the expression patterns of 12 candidate genes involved in ester biosynthesis in ‘Red Clapp Favorite’ during postharvest storage. The error bar represents the average ± SE for three replicates. The observed statistical significance of the differences among various letters **(A–D)** at P < 0.05.

In order to further investigate the expression of these candidate genes involved in ester biosynthesis in European pear, we conducted qRT-PCR analysis to examine the relative expression levels of these 12 candidate genes during the postharvest storage stage of ‘Red Clapp Favorite’. The correlation analysis and firmness comparison were conducted simultaneously. The findings revealed that during the latter part of postharvest storage, ‘Red Clapp Favorite’ exhibited a more rapid decline in firmness when compared to ‘Doctor Jules Guyot’ (**Supplementary Figure S3**). Notably, only one gene demonstrated a significant positive correlation with firmness (**Supplementary Figure S2**). The results revealed slight differences in gene expression between ‘Red Clapp Favorite’ and ‘Doctor Jules Guyot’ (**Figure 6**). Specifically, *PcLOX2*, *PcHPL1*, *PcFAD2*, *PcLIP2*, *PcLIP2-like*, *PcGLIP1* and *PcGLIP1-like* exhibited a gradual increase in expression with prolonged storage time until reaching a peak at 15 days before declining thereafter; whereas both *PcADH1* and *PcGLIP2* showed two cycles of initial increase followed by decrease during the storage period; furthermore, the expression of *PcFAD6* exhibited a decline over time in storage, whereas the expressions of *PcLOX1* and *PcLIP1* remained relatively stable. By combining the analysis of gene expression levels and transcriptome data of ‘Red Clapp Favorite’ pear and ‘Doctor Jules Guyot’ pear, it was found that *PcFAD2* and *PcLIP2* were the only two genes significantly positively correlated with high ester content in the transcriptome data. Additionally, only two genes, *PcFAD6* and *PcLIP1*, were significantly negatively correlated with high ester content. Meanwhile, in both ‘Doctor Jules Guyot’ pear and ‘Red Clapp Favorite’ pear, the relative expression levels of *PcFAD2* and *PcLIP2* showed an upward trend, but decreased after 15 to 20 days of storage post-harvest in ‘Red Clapp Favorite’ pear. *PcFAD6* showed down-regulated expression levels in both varieties, while the expression level of *PcLIP1* in ‘Red Clapp Favorite’ pear did not show significant changes.

## Discussion

The formation of the aroma profile in European pear fruit heavily relies on the synthesis of volatile compounds, which has also emerged as a significant driver for consumer purchase decisions. In fact, European pears need to go through a post-ripening process after picking to achieve the best edible quality and produce a strong fruit flavor (Goliáš et al., 2022; Liang et al., 2024). This research investigates and compares the volatile compound profiles of two distinct varieties of European pear during postharvest storage, revealing that both the type and content of these compounds undergo changes influenced by varietal characteristics and storage duration (**Figure 1**). The volatile compounds hexyl alcohol, butanol, hexyl aldehyde, 2-hexenal, hexyl acetate, amyl acetate, butyl acetate, and n-propyl acetate are commonly found in both cultivars and exceed 100 μg/kg during the duration of postharvest storage. The duration of storage shows a direct relationship with the levels of these compounds that potentially influencing the development of the fragrance characteristics in European pear (**Figures 2B, C**). Compared to ‘Doctor Jules Guyot’, ‘Red Clapp Favorite’ demonstrated elevated concentrations of volatile compounds such as alcohols, aldehydes, and acids during the late stage of ripening. Specifically, there was an increased presence of hexyl alcohol, butanol, hexal aldehyde, and 2-hexenal. Hexyl alcohol and 2-hexenal are well-known green leaf volatiles appreciated for their fragrant characteristics reminiscent of grass and fruit (Both et al., 2016; Chiam et al., 2022). This characteristic is also observed in Korla fragrant pears (*Pyrus sinkiangensis* Yu), a hybrid variety combining European and Oriental pear that emits a refreshing “fresh green” scent when fully ripe (Liu et al., 2019; Chen et al., 2020). Additionally, butanol typically imparts a wine-like flavor while ‘Red Clapp Favorite’ tend to display sweeter and more pronounced wine characteristics during extended storage. Additionally, there was an initial abundance of alkane compounds during storage in ‘Red Clapp Favorite’, which subsequently decreased over time. The presence of numerous alkane compounds indicates the fruit’s immaturity (Padilla-Jiménez et al., 2021), despite their lack of odor or only faint aroma. Nevertheless, it is imperative to acknowledge the significance of these compounds as intermediates in the synthesis of diverse volatile substances and should not be disregarded in the aroma development of European pear. Interestingly, esters are the predominant volatile compounds found in both ‘Red Clapp Favorite’ and ‘Doctor Jules Guyot’, significantly contributing to the overall scent characteristics of European pear—a finding aligns with earlier research (Chen et al., 2018). The esters exhibited a continuous accumulation trend during storage, reaching their peak levels in the later stages. ‘Doctor Jules Guyot’ demonstrated a faster rate of accumulation, particularly for hexyl acetate, amyl acetate, butyl acetate, and n-propyl acetate (**Figure 2B**). These compounds are typically associated with a pleasant and fruity olfactory profile (Ashwini and Virendra, 2017; Lu et al., 2017; Niu et al., 2019). Notably, hexyl acetate and butyl acetate accounted for 44% and 28% of the total esters found in ‘Doctor Jules Guyot’, respectively. The aroma formation of the European pear variety ‘Packham’s Triumph’ is influenced by several important volatile compounds, namely hexyl acetate, butyl acetate, and amyl acetate. Among these compounds, hexyl acetate plays the most significant role (Moya-León et al., 2006). Curuba (Passiflora mollissima (Kunth) L. H. Bailey) contains hexyl acetate and butyl acetate as its primary aroma constituents (Conde-Martínez et al., 2014). The esters butyl acetate, hexyl acetate, and 2-methylbutyl acetate are widely recognized as crucial components in apple fruit, playing a vital role in the development of its distinctive aroma (Dunemann et al., 2012). The findings of these studies are in line with our results, supporting the crucial contribution of ester compounds to the development of aroma in European pear. This discovery holds great value for enhancing our comprehension of the mechanism behind fruit aroma development in European pear.

The changes of esters in European pears during postharvest storage are intricate and diverse. Earlier research has established that the LOX pathway is primarily responsible for the biosynthesis of esters in apples, pears, and peaches (Schaffer et al., 2007; Zhang et al., 2011; Contreras et al., 2016; Luo et al., 2021b). Through transcriptome analysis, we have identified several genes that regulate ester biosynthesis (**Figure 3**) and subsequently correlated 12 candidate genes involved in ester biosynthesis with major ester compounds during ethylene production, fruit firmness, and storage period (**Figure 4**, **Supplementary Figure S2**). Among these genes, *PcFAD2* and *PcLIP2* exhibited a significant positive correlation with both ethylene production and ester compounds and both genes *PcFAD6* and *PcLIP1* show a significant negative correlation with ethylene and ester compounds. These two genes encode crucial enzymes FAD and LIP, respectively. In peaches, FADS has been shown to desaturate fatty acids to produce polyunsaturated fatty acids (Peng et al., 2023) while LIP is found to hydrolyze ester bonds to generate free fatty acids in tomatoes and certain bacteria (Li et al., 2020; Nisha et al., 2022). Hence, it is likely that *PcFAD2*, *PcFAD6*, *PcLIP1*, and *PcLIP2* may all accelerate the initial step of ‘Doctor Jules Guyot’ pear’s ester biosynthesis by encoding their respective enzymes, ultimately resulting in a rise in the content of esters in ‘Doctor Jules Guyot’.

By employing qRT-PCR, we acquired the relative expression levels of these 12 candidate genes during postharvest storage in ‘Doctor Jules Guyot’ and ‘Red Clapp Favorite’ (**Figures 5**, **6**). In ‘Doctor Jules Guyot’, the expression levels of *PcHPL1*, *PcADH1*, *PcFAD2*, *PcLIP2*, and *PcGLIP1* exhibited an ascending trend with the progression of postharvest storage duration, particularly noteworthy was the remarkable increase in *PcLIP2* expression by a factor of 17.5 within 10-15 days. The heightened expression of these genes aligns with previous investigations signifying their pivotal role in ester biosynthesis (Shi et al., 2018; Wu et al., 2022). However, contrary to our findings, peach demonstrated a declining pattern in HPL gene expression levels, likely attributed to varietal disparities (Cai et al., 2018; Meng et al., 2024). In addition, the expression levels of 9 genes in ‘Red Clapp Favorite’ exhibited a pattern of rapid growth (including *PcFAD2* and *PcLIP2*), surpassing that of ‘Doctor Jules Guyot’; however, they all demonstrated a subsequent decline after 15 days. By comparing the firmness, it was found that the ‘Red Clapp Favorite’ had a faster decrease in firmness than ‘Doctor Jules Guyot’ (**Supplementary Figure S3**). This may be one of the reasons why the expression level of the gene for lipid biosynthesis in ‘Red Clapp Favorite’ grows faster than that in ‘Doctor Jules Guyot’. Furthermore, the significant decrease in gene expression levels during storage and the fact that ‘Red Clapp Favorite’ contains fewer ester compounds compared to ‘Doctor Jules Guyot’ ultimately result in the total ester content of ‘Red Clapp Favorite’ being much lower than that of ‘Doctor Jules Guyot’ during storage (**Figures 1**, **2A, C**).

In summary, there are variations in volatile compounds among different varieties of European pear fruit during postharvest storage. These unique and intricate volatile compounds contribute to the distinct flavor characteristics of each variety and impact how consumers perceive and selection of flavor attributes, with esters being the main volatile compounds. Transcriptome analysis has identified genes involved in regulating ester biosynthesis, including *PcFAD2* and *PcLIP2* which were found to be highly expressed in both varieties. However, further investigation is needed to determine their specific biological functions in European pears. These findings suggest that candidate genes associated with volatile compound production, particularly ester biosynthesis, could be significant in improving the quality of European pear fruit.

## Conclusion

In summary, the volatile compounds formed during postharvest storage of European pear were analyzed and determined by GC-MS. The results showed that variations in the volatile compounds were observed among different varieties. However, ester compounds, particularly hexyl acetate, butyl acetate, amyl acetate, and n-propyl acetate, have been demonstrated to be significant factors influencing the aroma formation of European pear. Further investigations utilizing RNA-seq and qRT-PCR revealed the involvement of 12 genes in ester biosynthesis and regulation. Notably, *PcFAD2* and *PcLIP2* exhibited significant up-regulation and *PcFAD6* exhibits low expression levels in both varieties. Although low gene expression levels do not necessarily indicate low enzyme activity, genes associated with ester synthesis are likely to be upregulated. In conclusion, these findings offer novel insights into volatile production and ester biosynthesis during postharvest storage of European pear.

## Data Availability

The original contributions presented in the study are included in the article/**Supplementary Material**, Further inquiries can be directed to the corresponding author/s.

## Supplementary material

Supplementary Figure 1 Correlation analysis between RNA-seq data and real-time PCR.

Supplementary Figure 2 Correlation analysis of 12 ester biosynthesis candidate genes with firmness and major volatile esters of ‘Red Clapp Favorite’. Dark red indicates a positive correlation and dark blue indicates a negative correlation. The size of the circle indicates the degree of correlation. (**P<0.05, **P < 0.01* and ****P < 0.001*).

Supplementary Figure 3 Changes in firmness of ‘Doctor Jules Guyot’ and ‘Red Clapp Favorite’.

Supplementary Table 1 Primer sequences used in this study.

Supplementary Table 2 Summary of the findings from the GC-MS analysis.
