# Supplementary material for: Insights into the aroma volatiles and the changes of expression of ester biosynthesis candidate genes during postharvest storage of European pear
Source: Front Plant Sci. 2024 Nov 29;15:1498658. doi: 10.3389/fpls.2024.1498658 (PMC11638670; doi:10.3389/fpls.2024.1498658)
Supplement: Supplementary Figure 1 — Correlation analysis between RNA-seq data and real-time PCR. [file DataSheet1.doc]

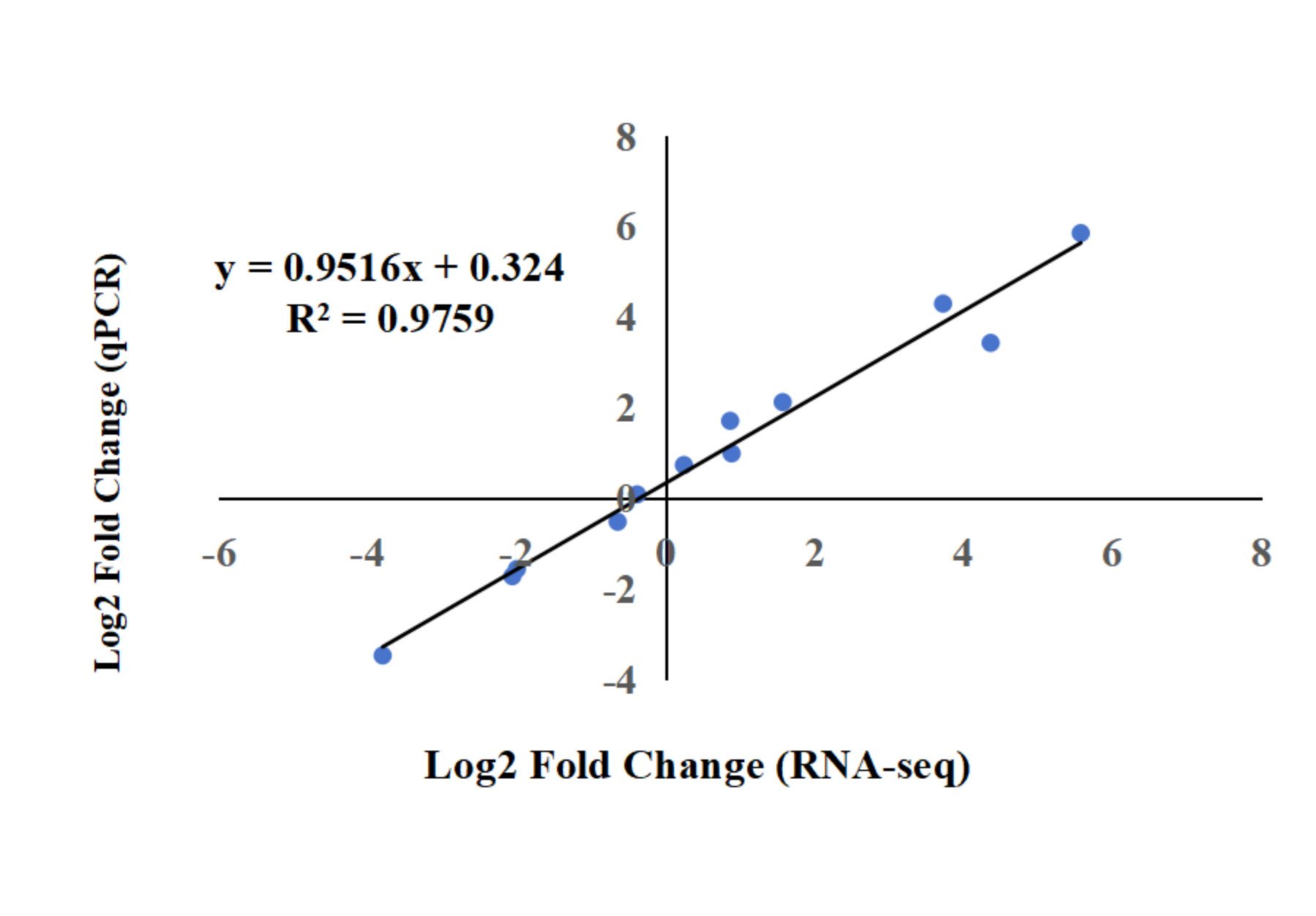


Figure S1. Correlation analysis between RNA-seq data and real-time PCR.


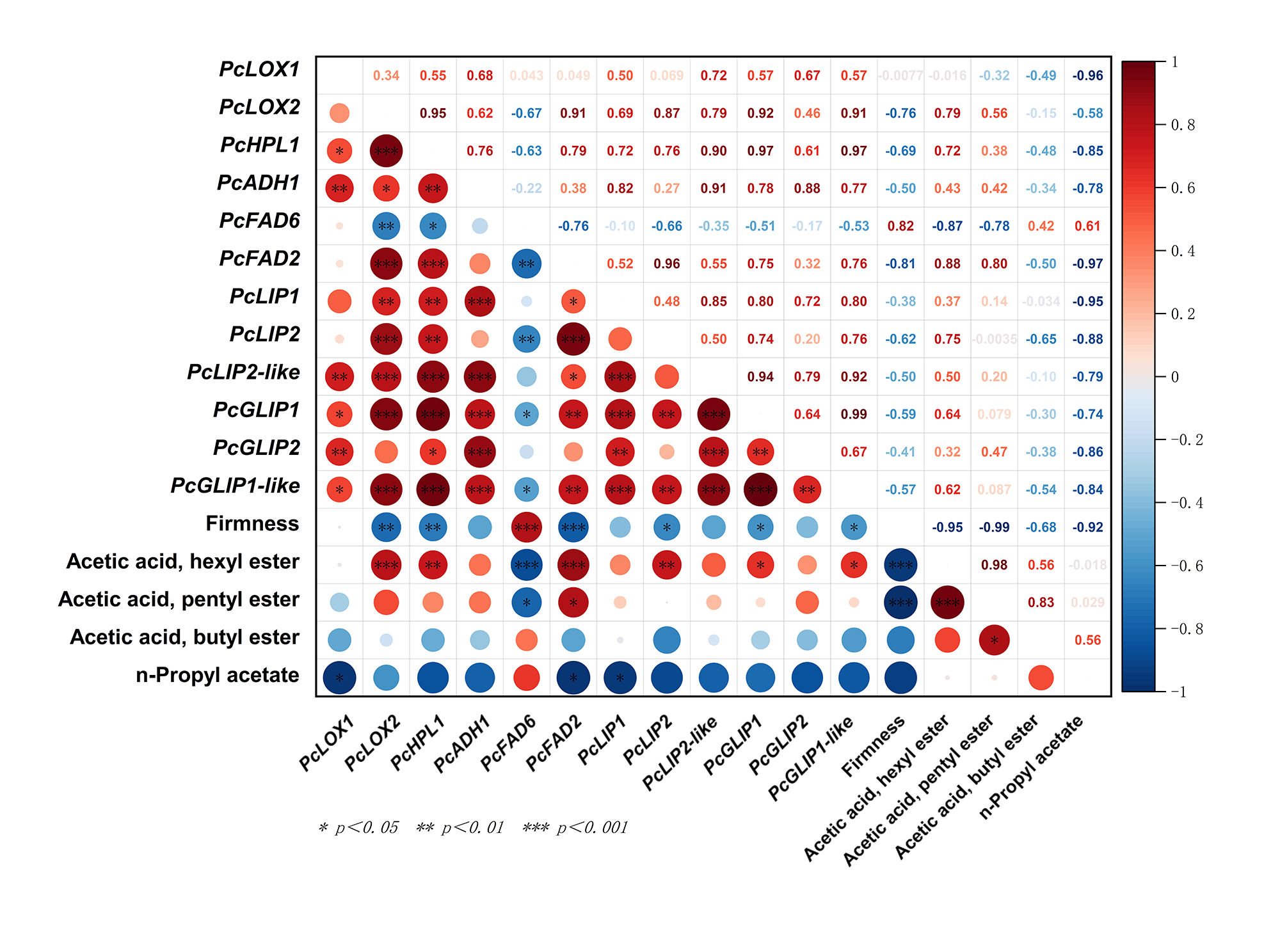


Figure S2. Correlation analysis of 12 ester biosynthesis candidate genes with hardness and major volatile esters of ‘Red Clapp Favorite’.


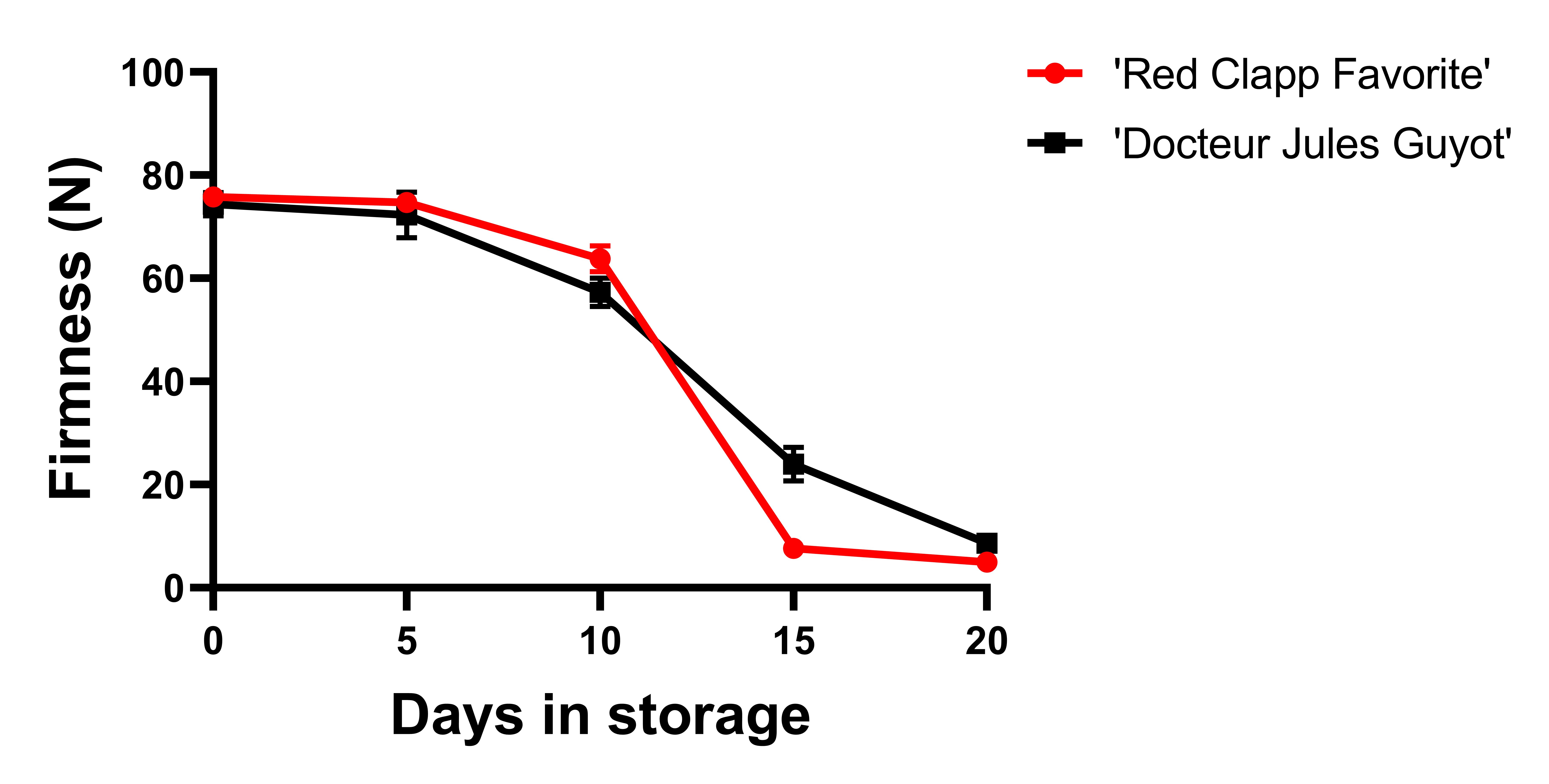


Figure S3. Changes in firmness of ‘Doctor Jules Guyot’ and ‘Red Clapp Favorite’.
