# Supplementary material for: Insights into the aroma volatiles and the changes of expression of ester biosynthesis candidate genes during postharvest storage of European pear
Source: Front Plant Sci. 2024 Nov 29;15:1498658. doi: 10.3389/fpls.2024.1498658 (PMC11638670; doi:10.3389/fpls.2024.1498658)
Supplement: Supplementary Table 1 — Primer sequences used in this study. [file Table1.docx]

**Supplementary**

Table S1 Primers sequences used in this study.

| Gene Name | Acession number | Primer Sequence (5’-3’) |
| --- | --- | --- |
| *PcLOX1*-F | Pbr004969.1 | GCTTCAAGGTTGGTGGACAG |
| *Pc**LOX1*-R |  | AAGTTCACTGCTGCATGGTG |
| *Pc**LOX2*-F | Pbr023784.2 | CCCCTCGATGACATTACCGA |
| *PcLOX2*-R |  | TACCTGAACAGCTCCAACGT |
| *Pc**HPL1*-F | Pbr041820.1 | GACACCTTTAAACCGGACCG |
| *PcHPL1*-R |  | ACCGGTCCCTGTGATTGAAT |
| *Pc**ADH1*-F | Pbr032936.1 | CCACGAGAAGCCAATCCAAC |
| *PcADH1*-R |  | CGGCTCTTGAAACCACCAAA |
| *Pc**FAD6*-F | Pbr034249.1 | GCGCCGCATATACCTTTCAA |
| *PcFAD6*-R |  | GCCAATTCCATGTAGCCTCG |
| *Pc**FAD2*-F | Pbr035830.1 | ATGCTGACACCCACAAGAGA |
| *PcFAD2*-R |  | ACACAGCCCTGAACATACCA |
| *PcLIP2*-F | Pbr014438.1 | AGTCTGCAAAGGATTCCGGA |
| *PcLIP2*-R |  | CTGAACCTGGTCCCTCTTGT |
| *PcGLIP1*-F | Pbr029061.1 | CTTGCCGCCTCTCAATGAAA |
| *PcGLIP1*-R |  | CACAACATGCCGTCTTTCCT |
| *Pc**LIP1*-F | Pbr012043.1 | TCCTTATCGGCCTCTTGACC |
| *PcLIP1*-R |  | TGTCTTCTCAAATCCCCGCT |
| *PcLIP2-like*-F | Pbr005182.1 | AGCTCGGGGTCCTTACTTTC |
| *PcLIP2-like*-R |  | TTGCATACCACCTTCCACCT |
| *PcGLIP2*-F | Pbr029062.1 | TCAAATTGCGGCGGAGAAAA |
| *PcGLIP2*-R |  | TGGACATTGTAAGGCCCTGT |
| *Pc**GLIP1-like-F* | Pbr029058.1 | AGGATGGTGGAGATGCAGTT |
| *PcGLIP1-like-R* |  | CATTTCATTGAGAGGCGGCA |
